# Intraoperative management of accelerated idioventricular rhythm triggered by tourniquet deflation: a case report and mechanistic insights

**DOI:** 10.1186/s12871-026-03911-y

**Published:** 2026-05-21

**Authors:** Jueheng Wu, Xiaohan Yang, Yalei Gao, Liang Guo, Fan Yang

**Affiliations:** 1 Department of Anesthesiology and Perioperative Medicine, The First Affiliated Hospital of Shandong First Medical University, Jinan, Shandong 250014 China; 2 Shandong Provincial Clinical Research Center for Anesthesiology, No. 16766 Jingshi Road, Jinan, Shandong 250014 China

**Keywords:** Accelerated idioventricular rhythm, Tourniquet shock syndrome, Reperfusion injury, Arrhythmia, Intraoperative complications

## Abstract

**Background:**

Accelerated idioventricular rhythm (AIVR) is a common reperfusion arrhythmia, but reports of AIVR triggered by tourniquet deflation in orthopedic trauma surgery are rare.

**Case presentation:**

A 59-year-old male with left tibiofibular open fracture underwent emergency open reduction and internal fixation. Recurrent AIVR and severe hemodynamic instability occurred twice immediately after tourniquet deflation, with wide QRS complexes, atrioventricular dissociation, and transient ST-segment depression, which was initially misdiagnosed as acute myocardial ischemia. The arrhythmia resolved rapidly after hemodynamic support and rewarming without antiarrhythmic drugs, and the patient recovered uneventfully.

**Conclusion:**

Tourniquet-induced AIVR is a multifactorial reperfusion-related arrhythmia that can easily be misdiagnosed as myocardial ischemia. It is usually self-limited and responds well to hemodynamic optimization. Strict tourniquet management and prompt differential diagnosis can improve perioperative safety in orthopedic surgery.

## Introduction

Pneumatic tourniquets serve as standard hemostatic instruments in orthopedic operations, yet the ischemia-reperfusion injury (IRI) induced by their release has garnered increasing clinical attention. Research indicates that lower limb ischemia exceeding 60 min results in a 3-5-fold elevation of plasma lactate, reactive oxygen species (ROS), and tumor necrosis factor-α (TNF-α) during reperfusion. These mediators heighten arrhythmia risks by disrupting myocardial calcium channel functionality and inducing autonomic nervous system dysregulation [1]. Notably, certain subtypes of AIVR typically present with ventricular rates between 50 and 100 beats per minute, mildly widened QRS complexes, and nonspecific ST-T alterations, which can be easily confused with myocardial ischemic events [1, 2]. Current understanding of the pathophysiological mechanisms underlying tourniquet-associated AIVR remains incomplete. This report elucidates the multifactorial mechanisms and clinical management principles through an in-depth analysis of a representative case. Although pneumatic tourniquets are essential for hemostasis in orthopedic surgeries, their use can lead to ischemia-reperfusion injury (IRI) upon release, which has been associated with arrhythmias, including accelerated idioventricular rhythm (AIVR).

## Case report

**Fig. 1:**
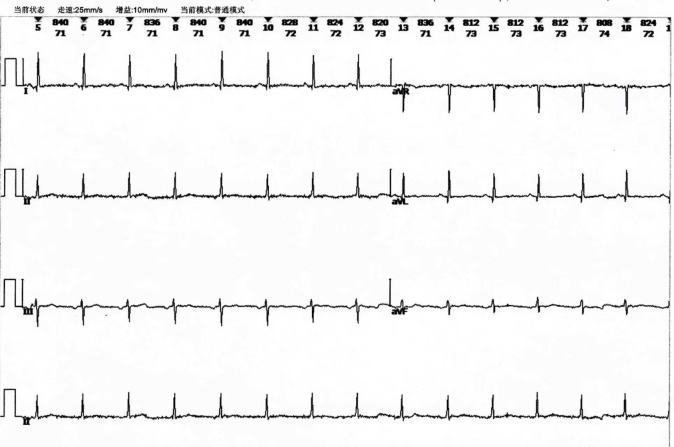
Preoperative baseline electrocardiogram showing sinus rhythm

A 59-year-old male construction worker underwent emergency internal fixation surgery for a Gustilo type II open fracture of the left tibia and fibula. His medical history included unmanaged hypertension for two years due to irregular medication adherence, without cardiovascular or metabolic comorbidities. Preoperative evaluation revealed a BMI of 25.4 kg/m², blood pressure of 148/92 mmHg, and unremarkable cardiopulmonary auscultation. Preoperative laboratory findings revealed serum potassium of 4.36 mmol/L, magnesium of 0.80 mmol/L, troponin-I of < 0.0061 ng/mL, and CK-MB of 3.2 ng/mL. Baseline electrocardiography demonstrated sinus rhythm (74 bpm) with flattened T waves in leads aVL and V5-V6 (Fig. 1), while transthoracic echocardiography was not performed due to the emergency nature of surgery and the patient’s stable postoperative course without recurrent arrhythmias.

General anesthesia was induced with midazolam 3 mg, etomidate 14 mg, cisatracurium 14 mg, and sufentanil 20 µg, maintained via continuous infusion of propofol (25 ml/h) and remifentanil (0.26 µg/kg/min). Intraoperative monitoring comprised continuous lead II electrocardiography, invasive arterial pressure measurement (via radial artery catheter placed after the initial arrhythmic event), bispectral index (BIS maintained at 40–60), and nasopharyngeal temperature tracking. A thigh pneumatic tourniquet (250 mmHg) was applied twice, with initial inflation lasting 50 min and subsequent inflation 60 min.

**Fig. 2:**
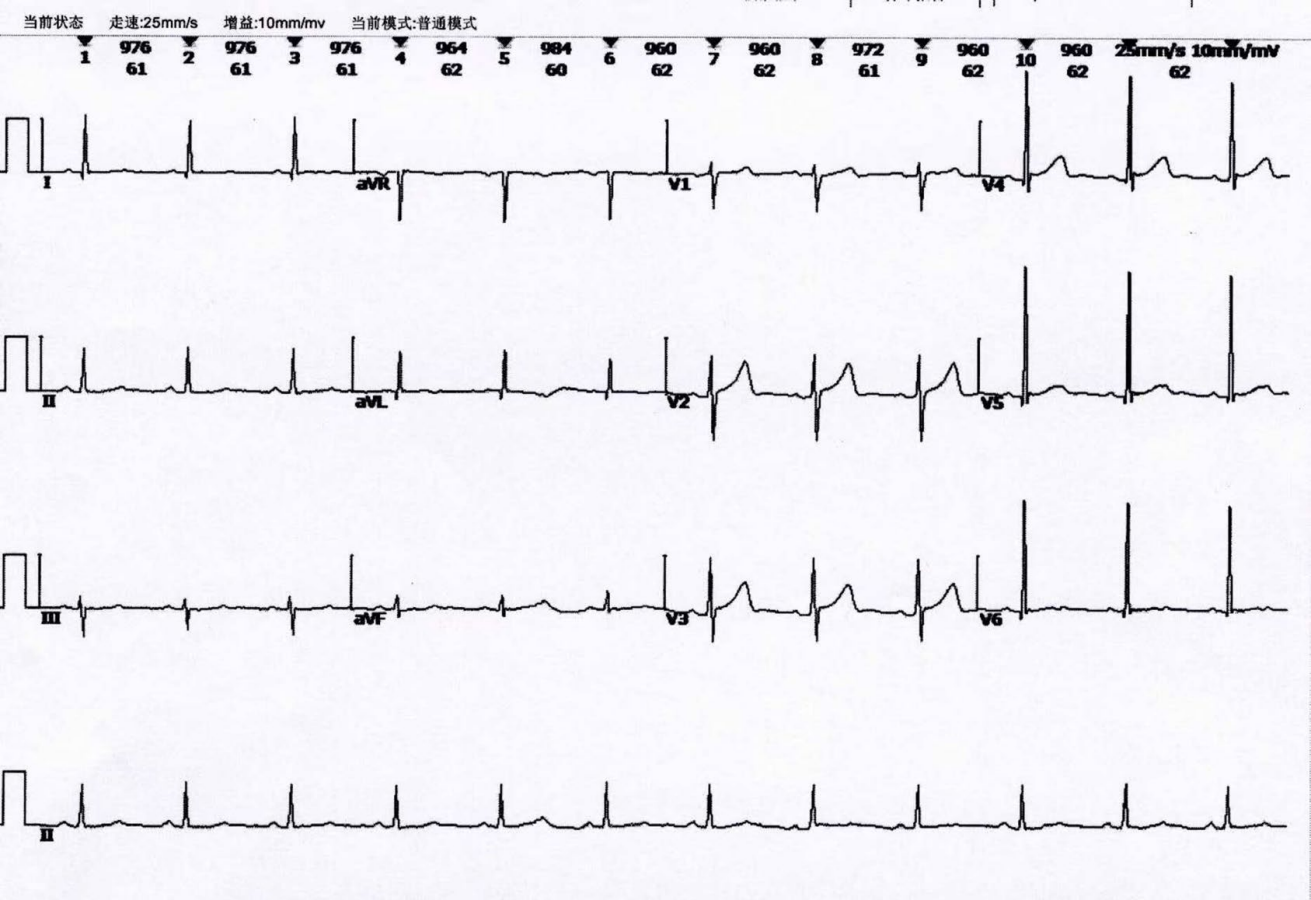
Twelve-lead electrocardiogram showing normal sinus rhythm after spontaneous resolution of the transient arrhythmia

During the first tourniquet deflation, the patient’s arterial blood pressure abruptly dropped from 125/78 mmHg to 90/50 mmHg as measured by non-invasive blood pressure monitoring. Electrocardiographic monitoring revealed wide QRS complexes (ventricular rate 58 bpm), complete atrioventricular dissociation, and ST-segment depression in lead II. Invasive arterial pressure monitoring via radial artery catheter was subsequently placed following the initial arrhythmic event. Immediate interventions included an intravenous bolus of norepinephrine 8 µg followed by continuous infusion (0.1 µg/kg/min) and nitroglycerin administration (0.5 µg/kg/min). Sinus rhythm was restored within three minutes, with blood pressure stabilizing at 110/65 mmHg. Recurrent transient ST-segment depression observed seven minutes later resolved spontaneously, and a subsequent 12-lead electrocardiogram showed no abnormalities (Fig. 2).

**Fig. 3:**
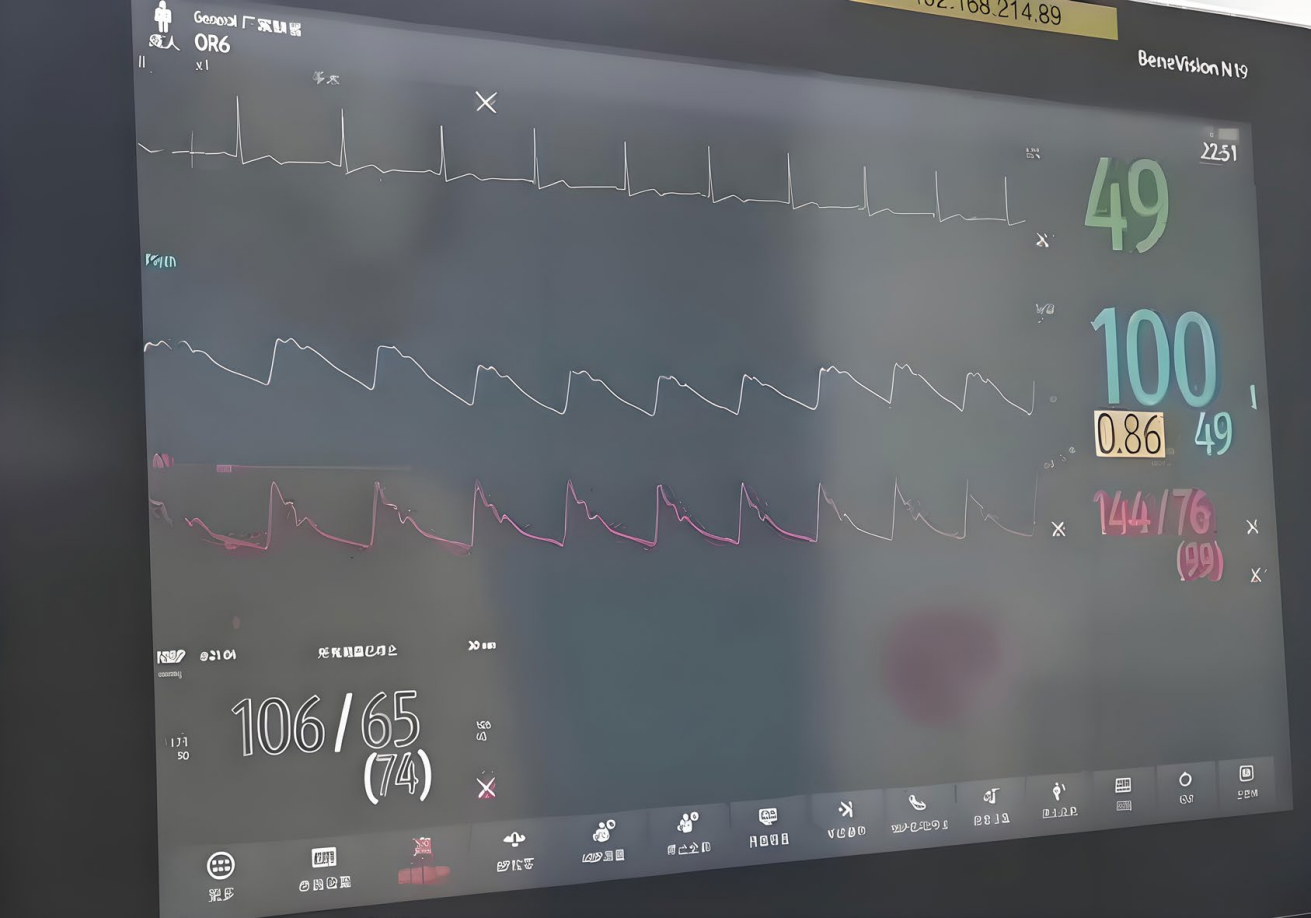
Pre-second deflation electrocardiogram

Prior to the second tourniquet deflation (30 min after the first), blood pressure measured 144/76 mmHg (Fig. 3).

**Fig. 4:**
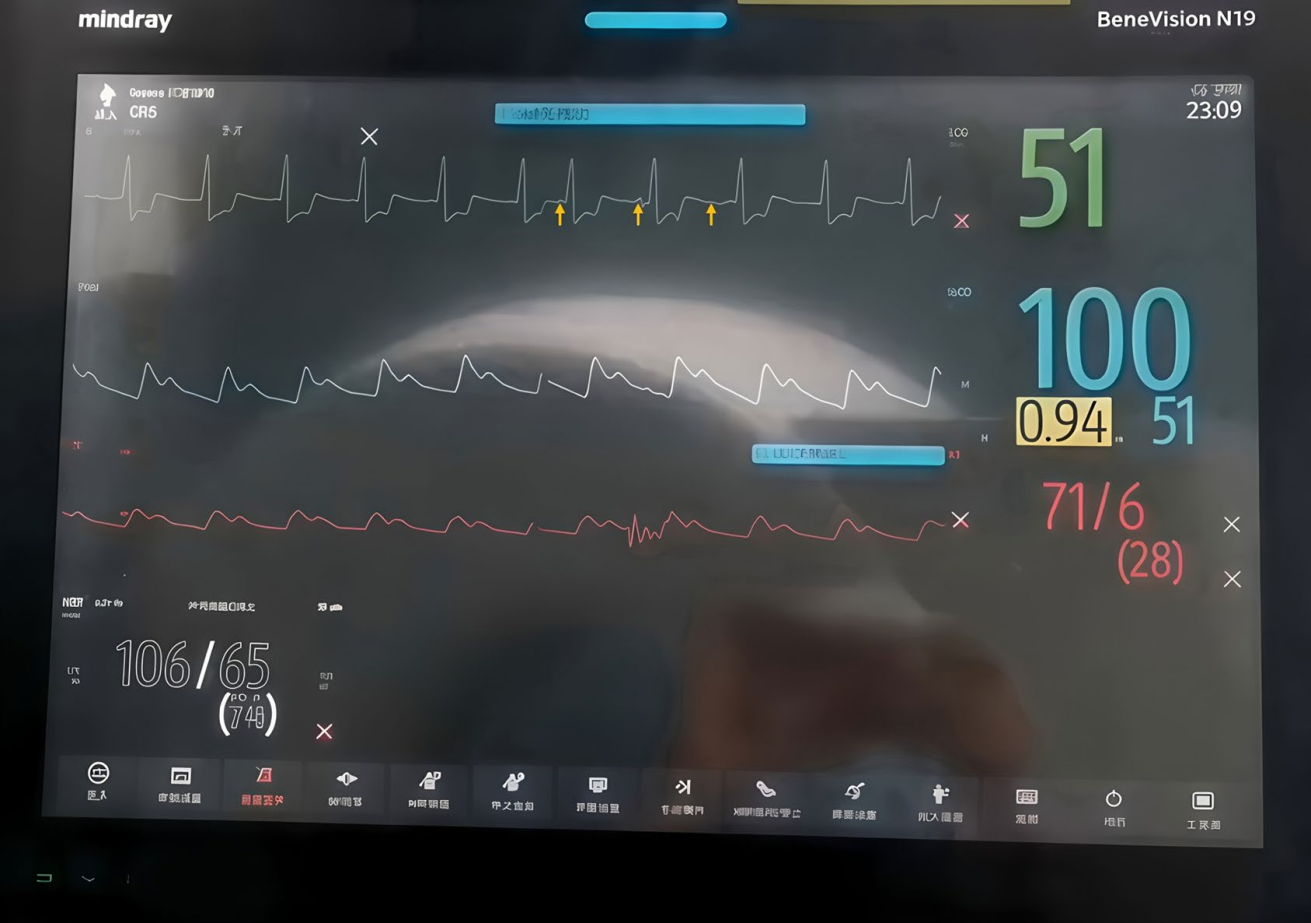
Active arrhythmia with marked ST-segment changes during the second tourniquet deflation. Black arrows indicate *P *waves

Upon deflation, arterial pressure precipitously decreased to 72/6 mmHg, accompanied by reappearance of wide QRS complexes and ST-segment depression (Fig. 4).

**Fig. 5:**
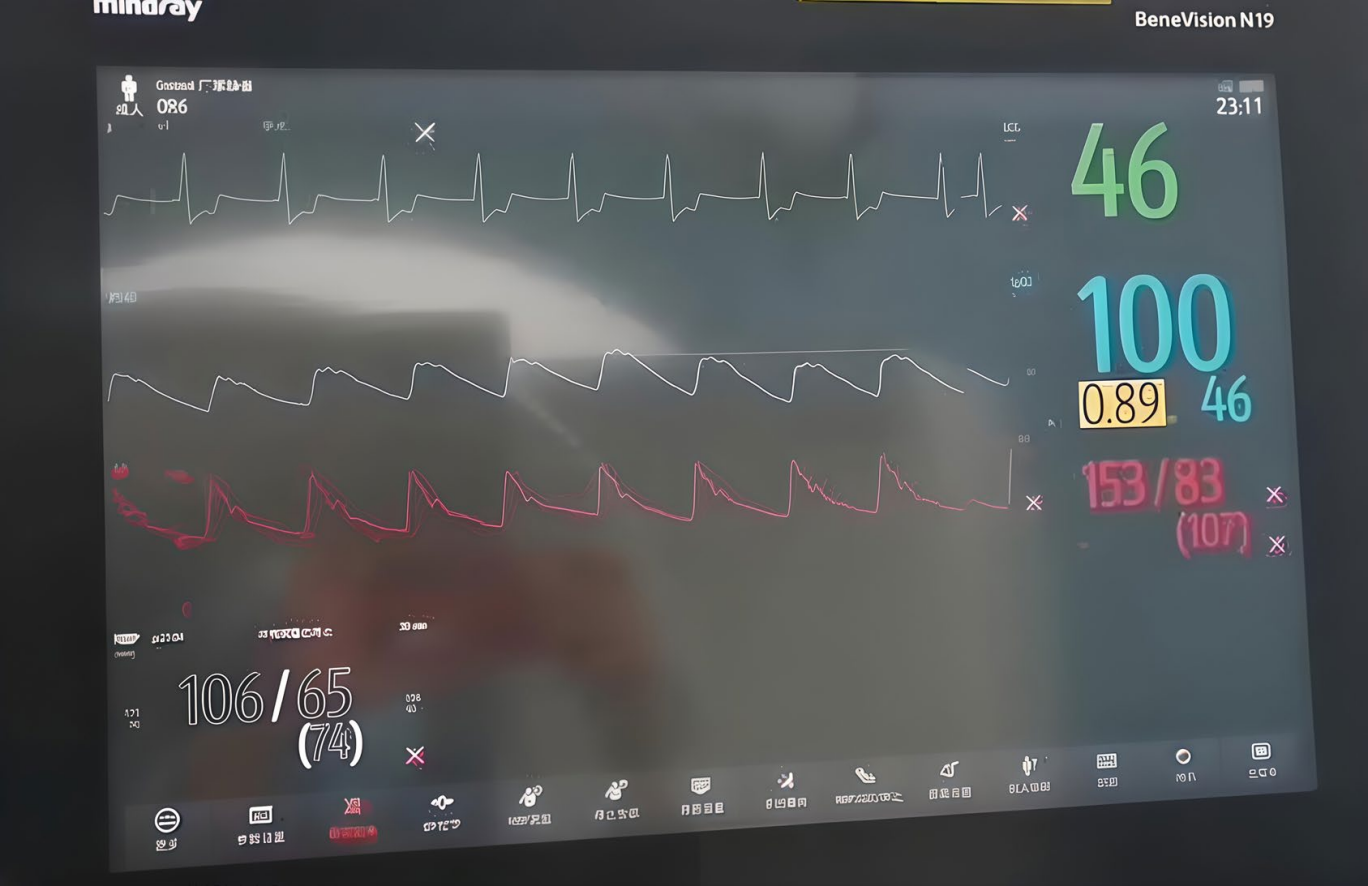
Dynamic QRS morphology shifts during hypotension

Emergency troponin-I testing confirmed levels < 0.006 ng/mL (CK-MB 2.01 U/L). Notably, dynamic alterations in QRS morphology correlated with blood pressure fluctuations were documented, with ventricular rates varying between 46 and 70 bpm and absence of consistent P waves (Fig. 5).

**Fig. 6:**
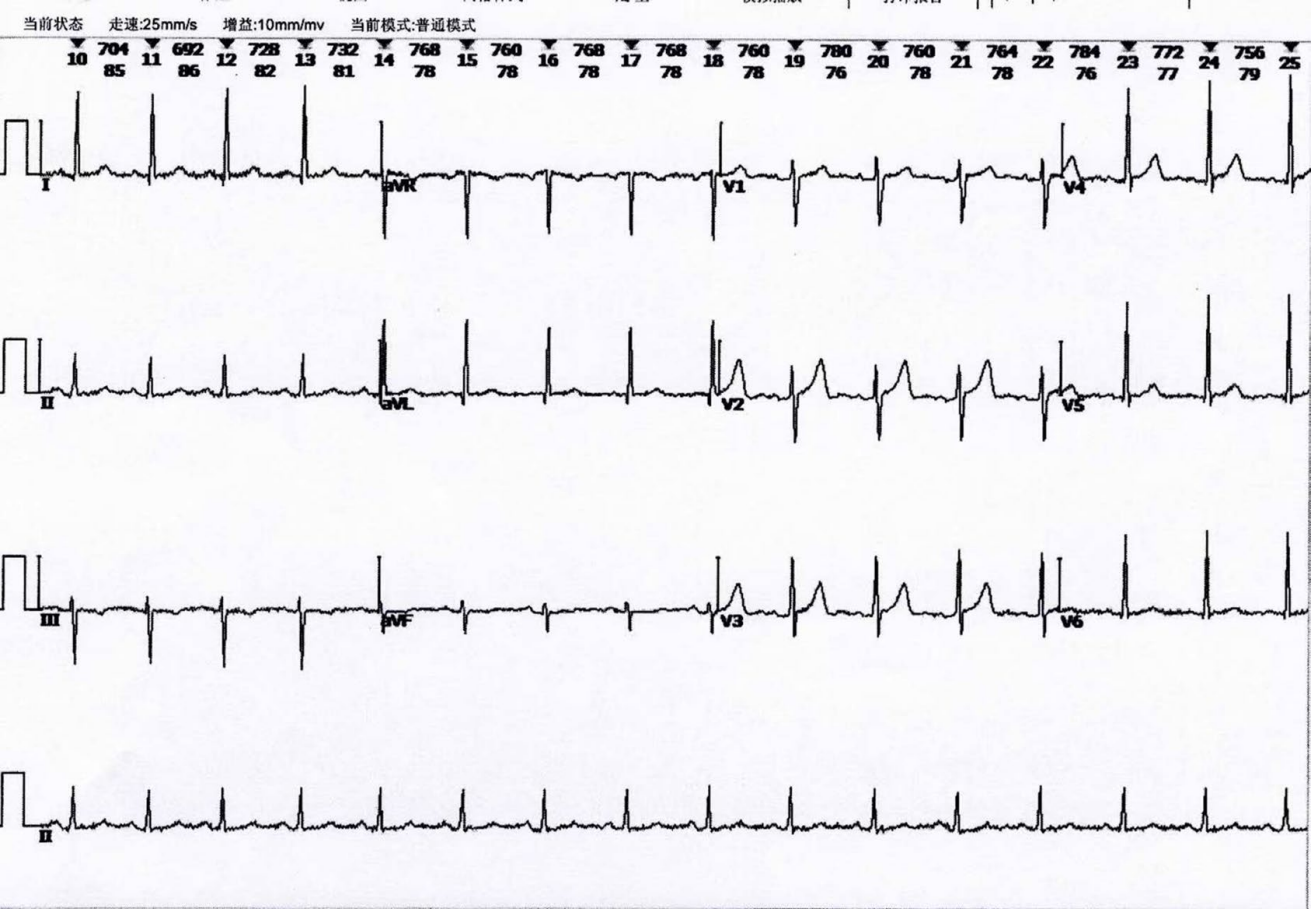
Postoperative electrocardiogram matching preoperative baseline findings

Without antiarrhythmic intervention, rhythm normalization occurred approximately four minutes after adjusting norepinephrine dosage and initiating passive rewarming. Postoperative extubation proceeded uneventfully, and a 24-hour ICU observation period revealed no recurrent arrhythmias. Follow-up electrocardiography reverted to preoperative findings (Fig. 6), with myocardial enzymes remaining within normal limits.

## Discussion

The AIVR episodes observed in this case may exemplify peripheral reperfusion injury-induced ventricular ectopy. Oxidative stress resulting from xanthine oxidase activation during reperfusion generates superoxide radicals, which may enhance ventricular automaticity through L-type calcium channel oxidation. This mechanism has been previously reported in studies on reperfusion arrhythmias [3, 4]. However, the precipitous drop in arterial pressure (144/76 mmHg to 72/6 mmHg) following the second tourniquet deflation suggests that hemodynamic factors may have played a pivotal role. This value likely represents genuine severe hypotension superimposed with measurement artifact (recent emergency catheter placement, potential transducer issues), evidenced by rapid clinical recovery. While tourniquet release typically induces transient hypotension, the magnitude of diastolic pressure reduction observed here (6 mmHg) indicates that the rhythm changes might primarily reflect low coronary perfusion pressure secondary to severe systemic hypotension, rather than purely biochemical reperfusion injury. The observed QRS morphology changes correlating with blood pressure fluctuations (Fig. 5) support this hemodynamic-dependent mechanism, as improved perfusion following norepinephrine administration coincided with rhythm normalization. Concurrently, intraoperative hypothermia (minimum core temperature 35.8 °C) likely suppressed sinoatrial node activity, as experimental data suggest an 8% reduction in depolarization rate per 1 °C temperature decline. This might have created conditions favoring a “slow-fast” rhythm competition between the sinus node and ectopic ventricular foci [5, 6]. Furthermore, hypothermia, electrolyte disturbances, and autonomic imbalance may also be involved in the pathogenesis of reperfusion arrhythmias. The patient’s low-normal serum magnesium level (0.80 mmol/L) may have compromised Na⁺/K⁺-ATPase function, predisposing to delayed repolarization and early afterdepolarizations [7]. Autonomic imbalance, characterized by sympathetic dominance during tourniquet inflation transitioning to vagal predominance post-deflation, may exacerbate sinus node suppression and ventricular automaticity [8].

Electrophysiologically, AIVR arising from proximal Purkinje fibers near the atrioventricular junction often manifests as relatively narrow QRS complexes (< 140 ms) [9–11], contrasting with the broader complexes (≥ 140 ms) associated with ventricular myocardial origins. In this patient, transient ST-segment depression in lead II likely stemmed from abnormal depolarization vectors rather than genuine myocardial ischemia, a phenomenon corroborated by the absence of troponin elevation and discordant response to nitroglycerin.

Differentiating AIVR from acute myocardial ischemia hinges on characteristic features: AIVR typically exhibits QRS duration ≥ 120 ms, atrioventricular dissociation, and ventricular rates exceeding atrial rates, whereas ischemic events preserve normal QRS morphology (barring preexisting conduction abnormalities) alongside persistent ST-segment deviations and biomarker elevations. This case underscores the diagnostic value of serial electrocardiographic analysis and biomarker profiling to avoid unnecessary interventions.

In retrospect, nitroglycerin was likely unnecessary and potentially exacerbated the hypotension, highlighting the need to confirm ischemia before empirical vasodilator use in tourniquet-induced arrhythmias.

A three-tiered management strategy is proposed: Preoperatively, strict adherence to tourniquet duration limits (≤ 90 min per cycle), magnesium supplementation to maintain levels ≥ 1.0 mmol/L, and active warming to sustain core temperature ≥ 36 °C are recommended. Intraoperatively, immediate exclusion of myocardial ischemia through 12-lead electrocardiography, troponin measurement, and echocardiography is paramount.

As AIVR is generally self-limiting and benign [12], therapeutic emphasis should focus on mitigating predisposing factors—coronary perfusion pressure maintenance, limb elevation to reduce the reperfusion burden [13], hemodynamic optimization, and active temperature correction. Postoperative vigilance remains critical, as delayed reperfusion arrhythmias may manifest up to six hours post-procedure.

Limitations: As this was an emergency surgery and the patient had good preoperative cardiac function, transthoracic echocardiography was not performed preoperatively. After surgery, the patient was extubated and transferred to the ICU without any complications, and cardiac ultrasonography was not conducted either. The lack of comprehensive cardiac evaluation limits the ability to definitively exclude underlying structural or ischemic heart disease, which represents the main limitation of this case report.

## Conclusion

Tourniquet-induced AIVR represents a clinically significant electrophysiological event arising from potential synergistic interactions among peripheral reperfusion injury, hypothermia, and electrolyte imbalances. Clinicians must recognize its hallmark features: temporal correlation with tourniquet use, transient ST-segment deviations, and favorable responsiveness to hemodynamic support. This case highlights the importance of considering AIVR in the differential diagnosis of transient arrhythmias following tourniquet deflation in orthopedic procedures, and adoption of a comprehensive “prevent-identify-support” framework can minimize diagnostic errors, reduce unnecessary pharmacotherapy, and enhance perioperative safety in orthopedic surgeries.

## Data Availability

No datasets were generated or analysed during the current study.

## Abbreviation

AIVR: Accelerated idioventricular rhythm

## References

[1] Dresen W, A.E.J.E.i.C M, Darby. Non-Sinus Rhythms with Normal Rates*.* 2020: pp. 169–172.

[2] Wang L, et al. Clinical characteristics and therapeutic strategy of frequent accelerated idioventricular rhythm. BMC Cardiovasc Disord*. *2021;21(1):425. 10.1186/s12872-021-02221-0 PMC8427942 34496747

[3] Miller FC, et al. Ventricular Arrhythm Dur reperfusion. 1986;112(5):928–32. 10.1016/0002-8703(86)90302-9 3776819

[4] Kam P, Kavanaugh R, Yoong FJA. The arterial tourniquet: pathophysiological consequences and anaesthetic implications. Anaesthesia*. *2001;56(6):534–45. 10.1046/j.1365-2044.2001.01982.x 11412159

[5] Hosseini S, et al. Hypothermia-induced accelerated idioventricular rhythm after cardiac surgery; a case report. BMC Cardiovasc Disord*. *2023;23(1):142. 10.1186/s12872-023-03178-y PMC10026505 36941559

[6] Akata T, et al. Changes in body temperature following deflation of limb pneumatic tourniquet. J Clin Anesth. 1998;10(1):17–22. 10.1016/s0952-8180(97)00214-6 9526932

[7] DiNicolantonio JJ, Liu J. J.O.h. O’Keefe. Magnesium for the prevention and treatment of cardiovascular disease. Open Heart. 2018;5(2):e000775. 10.1136/openhrt-2018-000775 PMC6045762 30018772

[8] Sato K, et al. Accelerated idioventricular rhythm following intraoral local anesthetic injection during general anesthesia. Anesthesia Progress. 2021;68(4):230–4. 10.2344/anpr-68-03-09 PMC8674851 34911065

[9] Nogami A et al. Purkinje-related ventricular tachycardia and ventricular fibrillation: solved and unsolved questions. JACC. Clinical Electrophysiology. 2023;9(10):2172–96. 10.1016/j.jacep.2023.05.040 37498247

[10] Gildea TH, J.T.J.T.P J, Levis. ECG diagnosis: Accelerated idioventricular rhythm. 2018;22:17–173. 10.7812/TPP/17-173 PMC5882183 29616912

[11] Jakkoju A, et al. Accelerated idioventricular rhythm. in Baylor University Medical Center Proceedings. 2018. Taylor & Francis. 10.1080/08998280.2018.1493323 PMC6499534 31080367

[12] Lemos M. M.F. da Mata, and I.C.J.C.i.t.Y. Mendes, Accelerated idioventricular rhythm in a healthy newborn: frightening but non-threatening. Cardiology in the Young. 2022;32(3):500–2. 10.1017/S1047951121003255 34365996

[13] Huang GS, et al. Bilateral passive leg raising attenuates and delays tourniquet deflation-induced hypotension and tachycardia under spinal anaesthesia: a randomised controlled trial. Eur J Anaesthesiol. 2014;31(1):15–22. 10.1097/EJA.0b013e32836286e3 23812622

